# Clinical Effectiveness and Efficacy of Chiropractic Spinal Manipulation for Spine Pain

**DOI:** 10.3389/fpain.2021.765921

**Published:** 2021-10-25

**Authors:** Carlos Gevers-Montoro, Benjamin Provencher, Martin Descarreaux, Arantxa Ortega de Mues, Mathieu Piché

**Affiliations:** ^1^Department of Anatomy, Université du Québec à Trois-Rivières, Trois-Rivières, QC, Canada; ^2^Cognition, Neurosciences, Affect et Comportement (CogNAC) Research Group, Université du Québec à Trois-Rivières, Trois-Rivières, QC, Canada; ^3^Madrid College of Chiropractic—Real Centro Universitario (RCU) María Cristina, San Lorenzo de El Escorial, Spain; ^4^GRAN Research Group, Université du Québec à Trois-Rivières, Trois-Rivières, QC, Canada

**Keywords:** low back pain, neck pain, spinal manipulative therapy, manual therapy, placebo

## Abstract

Spine pain is a highly prevalent condition affecting over 11% of the world's population. It is the single leading cause of activity limitation and ranks fourth in years lost to disability globally, representing a significant personal, social, and economic burden. For the vast majority of patients with back and neck pain, a specific pathology cannot be identified as the cause for their pain, which is then labeled as non-specific. In a growing proportion of these cases, pain persists beyond 3 months and is referred to as chronic primary back or neck pain. To decrease the global burden of spine pain, current data suggest that a conservative approach may be preferable. One of the conservative management options available is spinal manipulative therapy (SMT), the main intervention used by chiropractors and other manual therapists. The aim of this narrative review is to highlight the most relevant and up-to-date evidence on the effectiveness (as it compares to other interventions in more pragmatic settings) and efficacy (as it compares to inactive controls under highly controlled conditions) of SMT for the management of neck pain and low back pain. Additionally, a perspective on the current recommendations on SMT for spine pain and the needs for future research will be provided. In summary, SMT may be as effective as other recommended therapies for the management of non-specific and chronic primary spine pain, including standard medical care or physical therapy. Currently, SMT is recommended in combination with exercise for neck pain as part of a multimodal approach. It may also be recommended as a frontline intervention for low back pain. Despite some remaining discrepancies, current clinical practice guidelines almost universally recommend the use of SMT for spine pain. Due to the low quality of evidence, the efficacy of SMT compared with a placebo or no treatment remains uncertain. Therefore, future research is needed to clarify the specific effects of SMT to further validate this intervention. In addition, factors that predict these effects remain to be determined to target patients who are more likely to obtain positive outcomes from SMT.

## Background

Pain affecting the spine not only has a significant impact on the individual's health and functional ability but also carries considerable costs to the economy and society at large, mostly derived from treatment expenses and work absenteeism (1, 2). Back and neck pain combined are the number one cause of years lived with disability and the fourth leading cause of years lost to disability globally (2, 3). At any time, over 11% of the world population suffers from pain in the spine (4, 5). The prevalence has been increasing over the past decade (2), particularly among working-age females in high-income countries (5, 6). Chronic cases where pain lasts for more than 3 months significantly contribute to the increasing burden of spine pain (1, 2). Likewise, pain affecting the spine affects more than 50% of patients with chronic pain (1, 7), a condition whose estimated direct and indirect costs are hundreds of billions of dollars (8). The frequent use of inappropriate and invasive clinical interventions has been suggested as one of the main reasons for this increasing burden (1, 8, 9).

Throughout the past decade, recommendations for the evaluation and treatment of back pain have shifted toward less invasive, non-pharmacologic approaches. This is partly the consequence of the opioid use epidemic in North America, largely driven by high rates and doses of opioid prescriptions for non-cancer pain (10–12). The *Lancet* series on low back pain (LBP) highlighted an overreliance on secondary care, imaging, opioids, spinal injections, and surgery (9, 13). Instead, currently available data provide stronger support for the use of conservative interventions and self-management strategies (9, 13–15). This is reflected in the recent publication of systematic reviews and clinical practice guidelines exclusively devoted to summarizing the evidence and recommendations for non-invasive treatments for neck pain (NP) and LBP (16–18). Among these interventions, manual therapy is frequently recommended as one of many front-line options for spine pain (13–19).

Chiropractic is a health care profession concerned with the management of neuromusculoskeletal conditions and, more specifically, disorders affecting the spine (20). Arguably, chiropractors' area of expertise lies within the field of spine care and in the application of manual therapy (21, 22). Most chiropractic patients seek care for spine-related conditions (23–25). Likewise, people with back pain frequently visit chiropractors in high-income countries (23, 26, 27). Chiropractors strongly rely on the use of manual therapy, particularly spinal manipulation (SM), which is the main form of care they provide (24, 26). In the United States, where data are available, chiropractors perform a large proportion of all SM treatments (28, 29). Chiropractic SM is sometimes referred to as a chiropractic or spinal adjustment in the literature (30). Typically, a spinal adjustment consists of the application of a high-velocity, low-amplitude controlled thrust force to a spinal segment. For the purpose of this review, all interventions relying on the application of such thrust forces to the spine will be considered under the common terms SM and SMT (spinal manipulative therapy). The clinical indication of chiropractic SM has been the subject of controversy (31). However, SM provided by chiropractors for spine pain was recently demonstrated to be cost-effective and rarely inappropriate (32, 33). Furthermore, accumulating evidence on the effectiveness of SMT for the treatment of acute and chronic back and neck pain has rendered it an acceptable management option (8, 27).

Recent research on SMT suggests that chiropractic care may be evolving from the field of complementary and alternative medicine toward becoming a mainstream option for spine pain (22, 34). However, there is a need to summarize the most up-to-date research in the field for a better understanding of this evolution. Here, we aimed to review the most recent randomized clinical trials on the effectiveness and efficacy of SM and SMT for the management of NP and LBP, mostly published in the past decade. In addition, recommendations from state-of-the-art clinical practice guidelines will be presented, as well as a perspective on challenges and future directions for research on chiropractic SMT and spine pain. While the narrative review will be informed not exclusively by studies where chiropractors apply SM, this is done to inform chiropractic clinical practice with the best current available evidence.

## Methods

For the purpose of this review, the literature search was limited to SMT and manual therapy, when it comprised SM. Studies were included if they concerned the effectiveness and efficacy of SM, with no selection criteria for the professionals performing the intervention. Among these studies, only those published in English language between January 1st, 2009 and October 1st, 2019 were considered during the original selection. Relevant studies published after 2019 were added to the original selection during the publication process.

The following Databases were searched: Pubmed or Medline, Cochrane, CINAHL and the Index to Chiropractic Literature (ICL). The key search terms used for efficacy and effectiveness studies were: “spinal manipulation,” “spinal manipulative therapy,” “manual therapy,” “chiropractic” AND “efficacy,” or “effectiveness.” The results were filtered, and articles were selected with the key terms “lumbar” or “low back.” Since most studies concerned the lumbar spine, the terms “cervical,” “neck,” and “thoracic” were added to search literature on neck pain.

To narrow the search in line with the research question, clinical studies on the shoulder, upper extremity, chest pain, headache, dizziness, fibromyalgia, dysmenorrhea, or visceral conditions were excluded. Studies on pediatric populations were also excluded. The selection only included randomized controlled trials, systematic reviews, and clinical practice guidelines. Relevant articles were screened using the title and abstract. Two reviewers performed the search independently using these same criteria. After duplicates were eliminated, disagreements about inclusion were resolved through discussion and consensus.

A distinction needs to be made between effectiveness and efficacy, as these concepts refer to different levels of clinical evidence for an intervention (35). Effectiveness studies assess the outcomes of a treatment usually under circumstances that more closely resemble clinical practice. To do so, the intervention is commonly compared to another active treatment, such as standard care provided for the condition investigated (35). In contrast, efficacy studies are usually conceived as randomized clinical trials that are run under ideal and highly controlled experimental conditions. The treatment to be explored is preferably compared to an inactive comparator with known inertness, such as a sham or placebo (35). The most up-to-date evidence regarding the effectiveness of SMT for spine pain will be reviewed first, followed by a presentation of studies discussing its efficacy below.

## Effectiveness of Spinal Manipulative Therapy for Neck Pain

**Figure 1:**
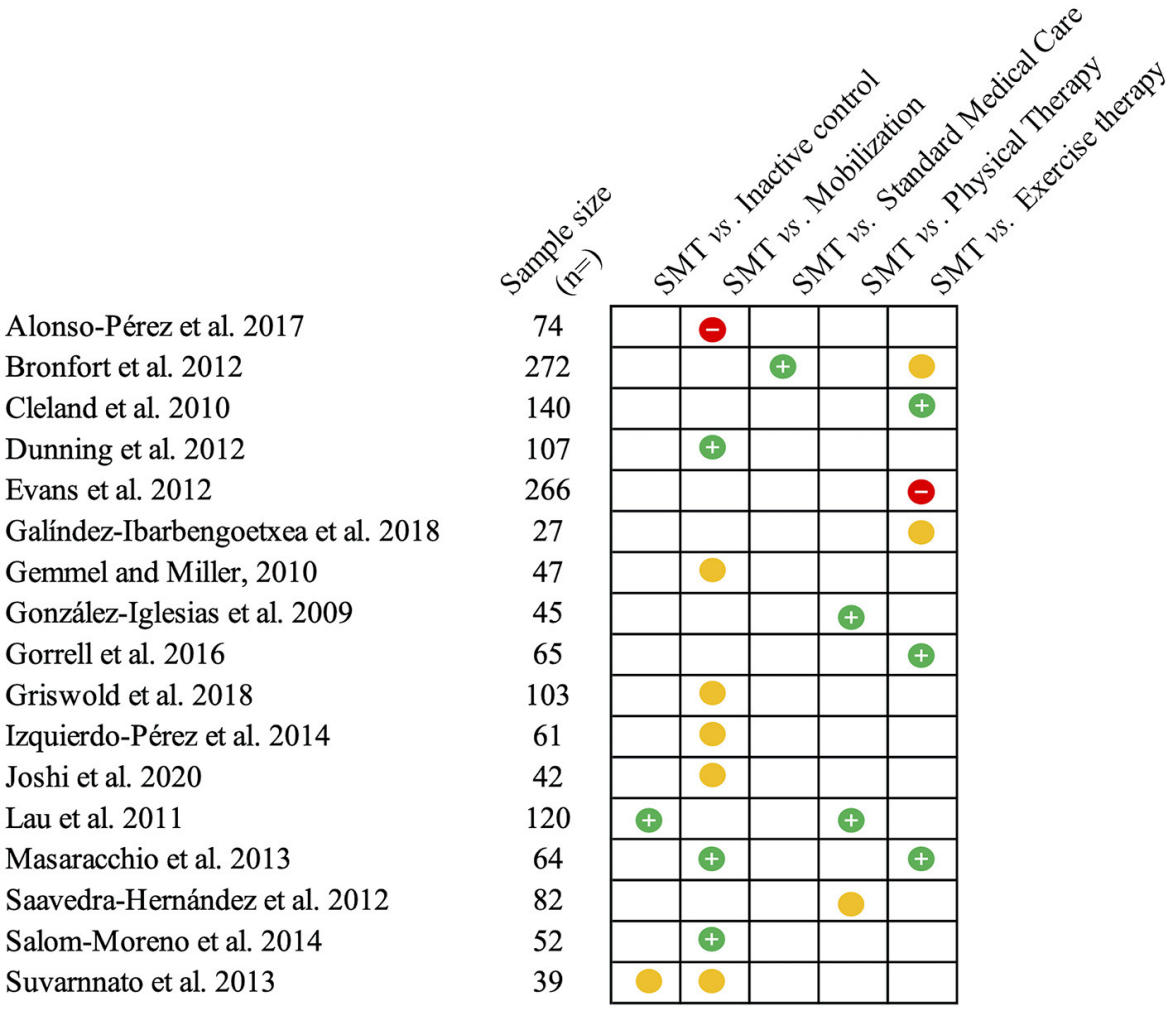
Summary of the studies reviewed on neck pain. This figure summarizes the main findings from the studies presented on the efficacy (compared to inactive controls) and effectiveness of spinal manipulative therapy (SMT) against different comparators for acute and chronic neck pain. The green circles with the positive sign indicate studies reporting pain-related outcomes in favor of SMT against or when added to the comparator. Yellow circles indicate similar effectiveness. Red circles with a negative sign indicate that SMT is inferior or does not add any value to the comparator.

Nonspecific NP is defined as pain between the skull and the first thoracic vertebra in the absence of a specific pathology or neurological sign (36, 37). Most cases of NP have been described as being of mechanical origin (38), which categorizes them as non-specific (36). In at least 10% of patients, non-specific symptoms persist beyond 3 months and can become chronic (38). In these cases, the condition is now defined as chronic primary (neck) pain (39, 40). The effectiveness of SMT has been examined in several studies on chronic primary NP as well as on acute and subacute non-specific NP. Most studies aimed to compare the effectiveness of a treatment based on SM to another active treatment, while fewer data are available concerning the efficacy of SMT compared to placebo (37, 41, 42). The most frequent active comparators used against SMT were other interventions commonly used for the management of NP, such as exercise or physical therapy modalities (43–50). Additional studies compared the application of SM to that of mobilization techniques or examined the effect of different SM application sites (cervical *vs*. thoracic) (51–58). However, these trials often measured short-term effects after short periods of care, which may not be as informative to clinical practice. All studies assessed pain intensity, the main outcome of interest for the present review, as measured with a numerical rating scale (NRS) or a visual analog scale (VAS). The second outcome measure of interest is the level of disability caused by NP, more commonly measured by the neck disability index (NDI) or the Northwick Park Neck Pain Questionnaire (NPQ). Outcomes may be assessed at variable follow-up times according to the study design. For both NP and LBP, a follow-up period of 1 month or less is generally considered short-term, intermediate-term is ~6 months and long-term follow-up after 1 year (59, 60). Figure 1 provides an illustration of the main results from the studies that are discussed below.

### Effectiveness of Spinal Manipulation Compared to Mobilization for Neck Pain

SM and mobilization are usually differentiated based on distinct biomechanical parameters of the forces applied, more specifically the force amplitude and rate of application (61). Whereas, SM has been characterized as a high-velocity low amplitude thrust, mobilization techniques generally involve the application of a force to a region or specific joint with larger (but variable) amplitude and lower velocity, without the thrust force (59, 61). Hence, mobilization is sometimes referred to as non-thrust SM (55, 59). When directly comparing the application of SM to mobilization, several studies reported no significant differences in pain intensity, disability, range of motion or quality of life, although all outcomes improved significantly regardless of the intervention (41, 51, 54, 56–58). However, when comparing both interventions to a control (inactive treatment) group, neither was successful at reducing pain (41). Thus, it is not clear if the reported effects were specific to the interventions, as will be discussed in the section Efficacy of spinal manipulative therapy for low back and neck pain.

Not all studies have reported consistent results. For example, a combination of cervical and thoracic SM produced greater reductions in NP and disability compared to mobilization of the same regions (52). In another study, patients with chronic primary NP experienced larger reductions in pain intensity with thoracic SM compared to mobilization (55). Furthermore, adding two sessions of thoracic SM to cervical mobilization and a home exercise program yielded greater improvement in pain ratings and disability than mobilization and exercise alone (53). What these studies have in common is thoracic SM being included as part of the active treatment. In contrast, studies reporting no differences between SM and mobilization often assessed cervical SM specifically (51, 54, 56). This is consistent with the conclusions from a recent systematic review and meta-analysis that SM, when applied to the thoracic spine, has a significant effect on pain and disability compared to mobilization (42). It could be argued that only thoracic SMT has demonstrated superiority to mobilization in the short term for NP and disability (59). Overall, the current body of literature provides stronger support for thoracic rather than cervical SMT for the treatment of NP (59, 62–65), suggesting that the site of application could influence the effectiveness of SMT for NP.

### Effectiveness of Spinal Manipulative Therapy Compared to Usual Care for Neck Pain

To evaluate the effectiveness of SMT for NP, outcomes are frequently compared to those of usual care. Usual care for NP has not been readily defined in the literature and could refer to one of two different approaches: standard medical care based on medication, home exercise and advice, or the application of standard physical therapy modalities including supervised exercise (42, 46). Two clinical trials compared the addition of SMT to a standard physical therapy treatment (electric or thermal stimulations, with or without educational material) for the management of acute (43) and chronic NP (45). In both cases, adding thoracic SMT provided greater reductions in pain intensity and disability lasting up to 6 months (43, 45). Interestingly, one session of cervical SMT did not prove to be more effective than Kinesio taping for NP, an approach frequently used in physical therapy practice (48). This may be interpreted as further evidence indicative of cervical SM being inferior to thoracic SM, although the evidence for this comparison is still scarce to draw inferences (42).

In patients with acute and subacute NP, one trial compared SMT against medication (acetaminophen, non-steroidal anti-inflammatory drugs or both) or a home exercise program with advice (46). The results from this study suggest that SMT is more effective than medication but not home exercise (46). Along the same lines, no between-group differences in pain and disability were reported 1 week after a home exercise program or a single session of SMT for patients with chronic NP (50). These data suggest that SMT is not superior to home exercise, although they do not allow us to determine whether SMT provides any additional benefit to exercise therapy. The addition of a single session of manual SM (as opposed to instrumental SM) to a stretching exercise program (used as a control intervention) was more effective in reducing NP intensity than the control exercise program alone (49). Similar results were found when two sessions of thoracic SM were added to an exercise program, partially assisted by a physical therapist and partially performed at home (44). These findings may indicate that one or two sessions of SMT may add value to exercise therapy for NP in the short term. However, in the long term, supervised exercise with and without SMT was found to be superior to a home exercise program for decreasing chronic NP intensity (47). Noteworthy, both studies assessing the effectiveness of multiple SMT sessions (>12) showed no superior benefit of SMT compared to exercise for NP of any duration (46, 47). These findings suggest that SMT does not provide additional benefits to certain forms of exercise in the longer term. In addition, they raise questions regarding the number of SMT sessions needed to influence NP outcomes. The available data do not indicate that a higher number of visits influences NP intensity, although this has only been studied as a secondary outcome in studies where cervicogenic headaches was the primary outcome (66, 67). It also remains to be clarified whether greater benefits are achieved with supervised or unsupervised exercise (as in a home exercise program) compared to SMT. Thus far, it has not been possible to identify one form of exercise that is superior to another for NP (68). Therefore, the results from systematic reviews of the past decade aiming to reconcile these discrepancies are discussed below.

Two earlier reviews examined the effectiveness of adding manual therapy (including SMT) to exercise as a single modal intervention or combined with other physical therapy modalities (69, 70). The addition of manual therapy to exercise provided greater short-term pain relief (70) and improved patient satisfaction (69) when compared to exercise alone in acute NP. However, subsequent reviews updated with newer data reached opposite conclusions on this question (71, 72). The meta-analysis by Fredin and Loras suggested that adding manual therapy (including SMT in 4/7 studies included) to exercise therapy does not result in additional clinical benefits (71). In contrast, Hidalgo et al. found moderate to strong evidence in favor of combining SMT and exercise for NP when compared to either of them alone (72). The most recent systematic reviews and meta-analyses examined the effectiveness of SMT by directly comparing it with usual management options (37, 42). Both reviews concluded that SMT is an equally effective approach to reduce pain and disability in the short term when compared to other interventions, including exercise (37, 42). Nevertheless, the strongest evidence was found in support of multimodal approaches, such as the combination of SMT and exercise (37).

Overall, the data reviewed indicate that SMT may be considered an effective intervention for the management of NP (73). Mobilization techniques seem to be comparable to SM, although some evidence suggests that thoracic SM may outrank mobilization. SMT is at least as effective as medication and physical therapy modalities for various stages of NP. The combination of SMT and exercise may provide one of the best approaches for the management of NP. These conclusions are summarized in Table 1.

**Table 1:** Effectiveness and efficacy (compared to inactive controls) of spinal manipulative therapy (SMT) for the management of neck pain (NP).

**Comparisons studied**	**Conclusions from previous studies**
SMT vs. inactive control	Inconsistent evidence that thoracic SMT may be superior to inactive treatment but not placebo^a^^−^*^d^*
SMT vs. mobilization	Evidence supporting thoracic SM (but not cervical) when compared to mobilization^c^^,^ ^e^^−^*^i^*
SMT vs. standard medical care	Insufficient evidence for a combination of cervical and thoracic SM when compared to analgesic medication and a home exercise program^j^
SMT vs. physical therapy	Evidence supporting SMT when compared to physical therapy^a^^,^ ^d^^,^ ^k^
SMT vs. exercise	Evidence supporting that SMT is not superior to exercise but may add value to unsupervised exercise^j^^,^ ^l^^−^*^n^*, unclear about supervised exercise^o^^,^ ^p^
Guidelines' recommendations	SMT is recommended after advice/patient education alone^q^, or in combination with exercise^r^^,^ ^s^. In acute NP, this combination may be offered before medication^s^

a *Lau et al. (45)*.

b *Suvarnatto et al. (41)*.

c *Gross et al. (59)*.

d *Coulter et al. (37)*.

e *Dunning et al. (52)*.

f *Saavedra-Hernández et al. (48)*.

g *Masaracchio et al. (53)*.

h *Salom-Moreno et al. (55)*.

i *Young et al. (65)*.

j *Bronfort et al. (46)*.

k *González-Iglesias et al. (43)*.

l *Cleland et al. (44)*.

m *Gorrell et al. (49)*.

n *Galíndez-Ibarbengoetxea et al. (50)*.

o *Evans et al. (47)*.

p *Masaracchio et al. (42)*.

q *Chou et al. (18)*.

r *Cote et al. (74)*.

s *Kjaer et al. (75)*.

## Effectiveness of Spinal Manipulative Therapy for Low Back Pain

**Figure 2:**
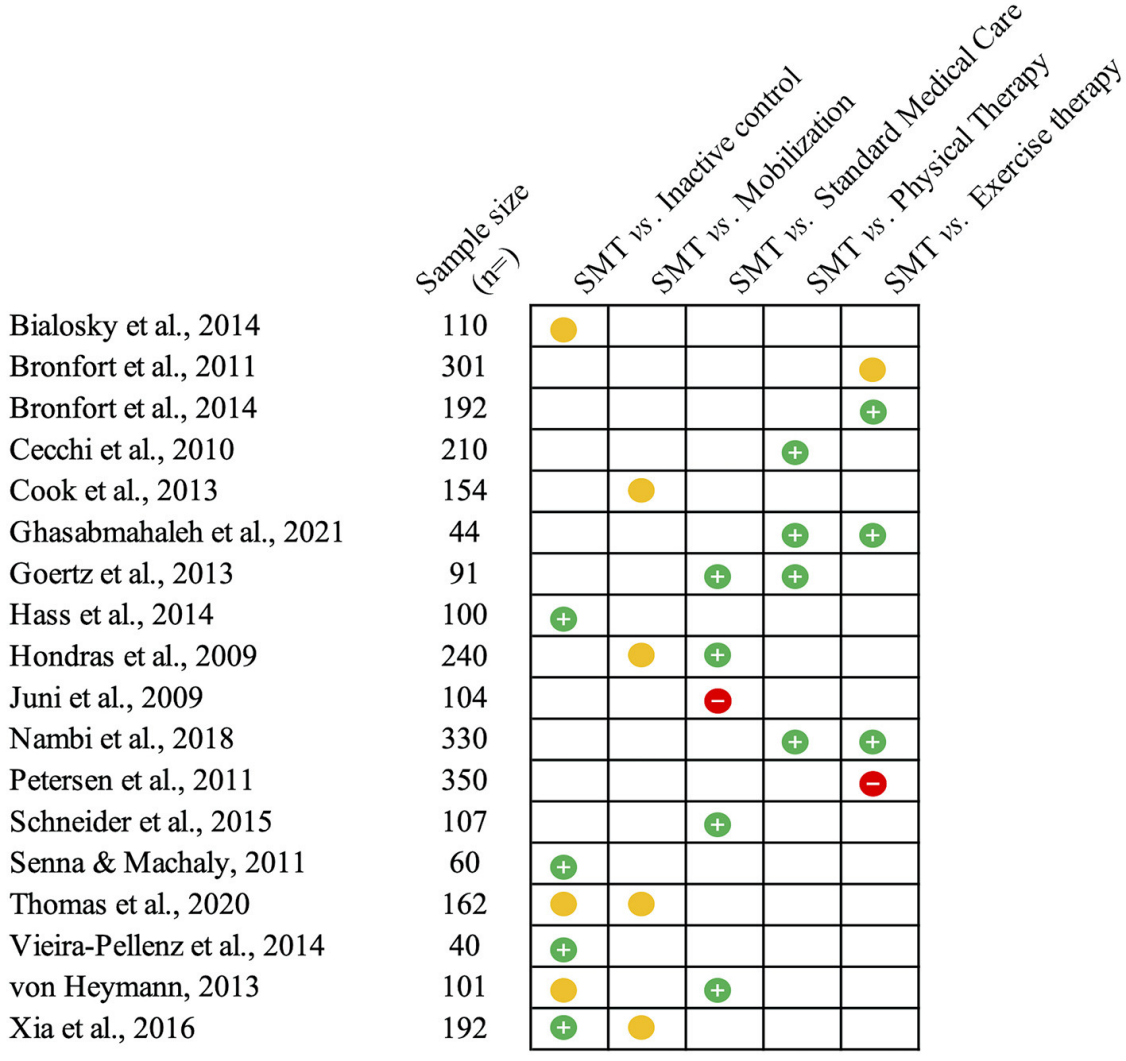
Summary of the studies reviewed on low back pain. This figure summarizes the main findings from the studies presented on the efficacy (compared to inactive controls) and effectiveness of spinal manipulative therapy (SMT) against different comparators for acute and chronic low back pain. The green circles with the positive sign indicate studies reporting pain-related outcomes in favor of SMT against or when added to the comparator. Yellow circles indicate similar effectiveness. Red circles with a negative sign indicate that SMT is inferior or does not add any value to the comparator.

LBP can originate from multiple musculoskeletal and neurovascular tissues, but for a large majority of cases, the specific structures involved remain elusive (76). Therefore, LBP presenting to primary care is predominantly considered non-specific, meaning that no specific source of nociception or pathology can be detected (76). When this condition persists or recurs beyond 3 months, cases are classified as chronic primary LBP (39, 76). Independent of duration, LBP is one of the most common complaints for patients presenting to primary care (77, 78). Hence, the effectiveness of SMT is frequently evaluated by comparing its application to standard medical care or physical therapy (79–87). Standard medical care based on medication is more frequently used during the early stages of LBP (79, 83, 85), while interventions based on exercise therapy are commonly prescribed for chronic primary LBP (81, 82, 86, 87). Fewer studies have examined the differences with sham/placebo interventions (88–93), and a handful have contrasted SMT to mobilization techniques for LBP (94–96). The outcome measures generally assessed include subjective reports of pain intensity and disability (the latter via the use of the Roland-Morris and Oswestry questionnaires), which are also the outcomes of interest for the present review. The main findings from the trials reviewed below are illustrated in Figure 2.

### Effectiveness of Spinal Manipulation Compared to Mobilization for Low Back Pain

A few studies have investigated the differences between SMT and mobilization for the management of LBP at different stages (94–96). Different mobilization techniques were employed, always consisting of the application of low-velocity forces of variable amplitude, without high-velocity thrust. Cook and colleagues recruited a sample of 149 patients with predominantly chronic LBP (symptom duration averaging >7 months) to examine the differences between thrust SMT and non-thrust mobilization in a pragmatic setting (95). No differences were found between groups, and more importantly, personal equipoises influenced pain and disability outcomes. In other words, different outcomes may be driven by practitioner preference for the technique (95). A specific mobilization technique where a flexion-distraction table is used to apply low-velocity forces was compared to SMT for subacute and chronic LBP (94, 96). No differences were reported between SMT and mobilization for any outcome, while both techniques were shown to be more effective than a waiting list for reducing pain and disability (96) and more effective than medication for disability (94). A recent systematic review reached the same conclusions regarding the equivalence of SMT and mobilization (60). For this reason, both techniques are often analyzed and recommended in guidelines as a single intervention (18, 97).

### Effectiveness of Spinal Manipulative Therapy Compared to Usual Care for Low Back Pain

Most clinical trials have examined the effectiveness of SMT for LBP by comparing SMT to another intervention recommended for its treatment (60). Standard medical treatment offered in primary care for LBP of recent onset has been used as an active comparator against SMT alone or as an addition to medical care (79, 83, 85). Standard medical care consisted of anti-inflammatory and analgesic medication, plus advice to maintain normal daily activity levels. In one of the studies, it was complemented with physical therapy modalities (83). When SMT was directly compared to usual medical care, patients receiving SMT reported significantly greater reductions in pain and disability at the 4-week follow-up (85). However, where SMT was provided in addition to standard care, the results were not consistent. Juni et al. reported no significant differences between groups in terms of pain reduction or use of analgesic medication after 2 weeks and 6 months (79). In contrast, Goertz et al. found that adding SMT significantly improved pain and disability at 2 and 4 weeks (83). These conflicting results could be explained by differences in the experimental designs. In particular, the number of SMT sessions delivered was not standardized among studies. Both trials applying a higher dose frequency (eight sessions in 4 weeks) observed a significant effect of SMT (83, 85). When a lower dose frequency of care was used (median of three SMT sessions in 2 weeks), no additional benefit of SMT was reported (79). Although SMT frequency might not have a significant impact on outcomes, increasing the frequency of visits in a few weeks showed a trend for decreasing both pain and disability (67). Frequency responses to SMT have not been assessed for early stages of LBP; therefore, a potential effect cannot be ruled out. It may be argued that three sessions (but not eight) of SMT may be insufficient to observe a significant effect. Conclusions from a recent meta-analysis provide support for the idea that SMT results in modest improvement in pain and function for acute LBP (98). The size of the benefit for pain was found to be approximately the same as that with non-steroidal anti-inflammatory drugs (reduction in 9.9 points for SMT vs. 8.4 points for anti-inflammatories, out of 100) (98). In light of these findings, it remains unclear whether SMT adds value to standard medical care for the management of acute and subacute LBP, although the limited evidence available suggests that both may be comparable.

For chronic stages of LBP, the response to SMT has more often been compared to physical therapy modalities, including exercise (80–82, 84, 86, 87). For chronic LBP-related leg pain (referred and radicular), two clinical trials observed that SMT added significant value to home exercise (84) and multimodal physical therapy, including exercise (87). After 12 weeks, both LBP, leg pain, and associated disability were significantly reduced when SMT was added to the active control treatments (84, 87). Adding SMT to exercise and laser therapy was also more effective than the provision of exercise alone or when combined with laser therapy for chronic LBP patients (86). The differences were maintained at the 12-month follow-up. In line with these results, a systematic review found moderate evidence to support the combination of SMT, exercise, and standard medical care for chronic LBP (99). Nevertheless, this does not allow us to determine how SMT directly compares to exercise.

A clinical trial examined the differences between SMT, back school (a combination of patient education and exercise), or physical therapy for patients with chronic LBP (80). The authors reported that SMT conveyed the largest reduction in disability at 6 months, and in both pain and disability after 1 year. Conversely, the direct comparison of SMT to a home exercise program or supervised exercise did not show any differences between interventions in pain or disability outcomes, neither in the short nor long term (81). Furthermore, a study allocated predominantly chronic LBP patients to receive either SMT or exercises derived from the McKenzie method, in addition to information and advice from the “Back book” (82). Both approaches resulted in clinically meaningful improvements, but the McKenzie method led to significantly larger improvements in disability after 2 and 12 months (82). It may be argued that different forms of exercise could have different effectiveness for chronic LBP and therefore compare differently with SMT. This hypothesis was rejected by a systematic review, which found that no form of exercise is superior to another for chronic LBP (100). More recently, these results were contradicted by a network meta-analysis reporting that Pilates, stabilization/motor control, resistance, and aerobic exercise are the most effective exercise approaches for LBP (101). Interestingly, McKenzie exercises were not found to be better than a true control. However, this must be interpreted with caution due to the low quality of the evidence available to date (101).

Multiple systematic reviews have examined the effectiveness of SMT (with or without mobilization) compared to exercise. Equivalent clinical benefits have been reported for both interventions in patients with both acute and chronic LBP (99, 102). A recent meta-analysis by Coulter et al. found moderate-quality evidence to suggest that SMT significantly reduces pain and disability in patients with chronic LBP compared to both exercise and physical therapy (97). A set of three meta-analyses investigated the effects of SMT in patients with chronic LBP by comparing SMT or mobilization to currently recommended therapies (mainly exercise), non-recommended or ineffective therapies (inactive controls), and a combination of interventions (60, 103, 104). The data pooled from 47 randomized controlled trials indicated that SMT provides improvements in pain and disability that are similar to those of recommended therapies for the management of chronic LBP, including exercise (60). The analysis of individual participant data from 21 of these trials confirmed these findings while not being able to identify any individual characteristic that could act as a moderator of the benefits provided by SMT (103, 104). Therefore, chronic LBP patients may benefit from SMT and exercise to a similar extent, although it is still not possible to determine which treatment approach will be more beneficial for which patients.

The presented data indicate that SMT conveys a therapeutic benefit at least as important as other standard and recommended approaches of care for LBP. Indeed, patient-centered outcomes of pain intensity and disability were found to respond similarly to SMT when compared to standard medical care or physical therapy (105). Interestingly, a review of pragmatic trials found that chiropractic care (always including SMT) was as effective as standard physical therapy (106). This design does not allow the drawing of inferences regarding the contribution of a specific intervention offered by chiropractors (i.e., SMT). Nonetheless, the results are consistent with fastidious studies comparing SMT to the same modalities, indicating that chiropractic SMT should be considered as effective as any other recommended intervention, particularly for chronic LBP. These conclusions are summarized in Table 2.

**Table 2:** Effectiveness and efficacy (compared to inactive controls) of spinal manipulative therapy (SMT) for the management of low back pain (LBP).

**Comparisons studied**	**Conclusions from previous studies**
SMT vs. inactive control	Insufficient evidence for SMT when compared to sham treatment^a^^−^*^j^*
SMT vs. mobilization	Evidence supporting that SMT and mobilization are equally effective^k^^−^*^n^*
SMT vs. standard medical care	Inconsistent evidence, only for acute LBP, could depend on dose^o^^−^*^r^*
SMT vs. physical therapy	Evidence supporting that SMT adds value to and is at least as effective as physical therapy for chronic LBP and leg pain^s^^−^*^w^*
SMT vs. exercise	Evidence supporting SMT being as effective as exercise; stronger evidence for chronic LBP^n^^,^ ^r^^,^ ^x^^−^*^z^*
Guidelines' recommendations	For acute and chronic LBP with or without leg pain, SMT is recommended alone^aa^^−^*^ac^* or more often as part of multimodal care along with advice, education, reassurance and exercice^ad^^−^*^ai^*.

a *Senna and Machaly. (88)*.

b *von Heymann et al. (89)*.

c *Bialosky et al. (90)*.

d *Bialosky et al. (90)*.

e *Vieira-Pellenz et al. (92)*.

f *Thomas et al. (93)*.

h *Scholten-Peeters et al. (107)*.

h *Ruddock et al. (108)*.

i *Gianola et al. (109)*.

j *Lavazza et al. (110)*.

k *Hondras et al. (94)*.

l *Cook et al. (95)*.

m *Xia et al. (96)*.

n *Rubinstein et al. (60)*.

o *Juni et al. (79)*.

p *Goertz et al. (83)*.

q *Schneider et al. (85)*.

r *Paige et al. (98)*.

s *Cecchi et al. (80)*.

t *Bronfort et al. (84)*.

u *Nambi et al. (86)*.

v *Ghasabmahaleh et al. (87)*.

w *Goertz et al. (105)*.

x *Bronfort et al. (81)*.

y *Hidalgo et al. (99)*.

z *Coulter et al. (97)*.

aa *Chou et al. (111)*.

ab *Qaseem et al. (17)*.

ac *Kirkwood et al. (15)*.

ad *Dagenais et al. (112)*.

ae *Bernstein et al. (113)*.

af *Wong et al. (114)*.

ag *Bussieres et al. (115)*.

ah *Stochkendahl et al. (116)*.

ai *Bailly et al. (19)*.

## Efficacy of Spinal Manipulative Therapy for Low Back and Neck Pain

Few studies have used inactive treatment to assess the efficacy of SMT for patients with NP, and those who had, mostly examined the immediate effects of a single SM, which may or may not provide relevant clinical information (37, 59). Adding SMT to standard care for one group and comparing the outcomes to those of the group only receiving standard care (43, 45, 53) could be interpreted as a comparison of SMT against no treatment (59). However, an ideal comparator should be inactive and effectively blind patients. This design is less common in research on spine pain overall, as sham procedures are rarely inert or otherwise unsuccessful in blinding patients (117). For SMT or manual therapy in general, this is further limited by the complexity of designing a sham that mimics SM but that produces little or no effect (118, 119). A graphic summary of the results from these studies is available in Figure 1.

A single thoracic SMT or mobilization was compared to a control consisting of manual contact held for 2 min (41). No differences between groups were found in NP intensity post-intervention, albeit significant increases in range of motion were observed after SMT. It is possible that participants were not successfully blinded, as this was not assessed (41). Moreover, patients likely had different expectations for SMT compared to the control intervention, which may have influenced outcomes. Indeed, expectations are known to be a reliable predictor of clinical pain treatment outcomes (120). Based on the fact that it only induces short-lasting superficial heating effects, infrared radiation might serve as a more suitable inactive control (45). Significant improvement in NP and disability was reported after thoracic SMT compared to this control. However, expectations of pain relief were likely very different for both interventions. These studies seem to confirm that the control procedures are heterogeneous and not always indistinguishable, which may result in inadequate blinding (121). The latest Cochrane review concluded that thoracic SMT, when compared to inactive treatment, led to significant reductions in pain intensity at short and intermediate term for early stages of NP and in disability at any stage (59). Notwithstanding, evidence favoring thoracic SMT specifically against placebo is scant (42). Therefore, the specific effects of SMT for NP when examined against placebo remain not well-understood.

Sham SMT has been more frequently explored as a placebo comparator in efficacy trials of SMT for LBP (108). It is common to use a similar hand placement and patient position for sham SM while applying biomechanically different forces (e.g., lower force or velocity, non-therapeutic direction, or point of application) or no force at all (88–90, 92). Figure 2 illustrates the direction of the findings for each of the studies discussed below.

The immediate efficacy of a single SM for LBP of unspecified duration was compared against a sham manipulation, positioning the patient but not applying any force (92). Patients reported immediate pain relief after SMT compared to sham; however, these results may or may not be transferable to the clinical setting. In the longer term, SMT was compared to diclofenac or placebo for acute LBP (89). The large rate of drop-out in the placebo group (11/25 subjects) compared to both treatment arms only allowed for comparisons between SMT and diclofenac (5/38 and 4/37, respectively), but may indicate the clinical superiority of both treatments over placebo (89). Interestingly, the placebo used was a “real” SM, although applied to a distant and “non-dysfunctional” segment (opposite sacroiliac joint). This placebo may have been successful at blinding patients but is not necessarily inert. In a clinical trial recruiting patients with LBP of any duration, no differences were found in clinical pain intensity and disability after SMT, placebo, or no treatment (90). This was despite patients experiencing a significant decrease in temporal summation of pain immediately after receiving the first SM. Changes in temporal summation have been found to highly correlate with clinical pain. The authors attributed the negative results to the fact that most recruited patients had chronic pain (duration > 12 weeks), which may be less likely to respond to SMT (90). Recent data do not necessarily support this hypothesis. The negative results may rather be explained by a period of treatment and follow-up that was likely too short (2 weeks) for patients with long pain duration (90). The efficacy of SMT for patients with chronic LBP was also examined over a longer period of time (10 months) (88). During the first month of treatment, two groups received the same SMT, and a third group was exposed to sham manipulation. After the first month, SMT performed better than the sham for pain and disability outcomes (88). Subsequently, one of the two SMT groups continued to receive maintained SMT (every 2 weeks) for 9 more months. The two remaining groups received no additional treatment. Upon completion of the study, continued exposure to SMT after the first month was significantly associated with lower pain and disability, suggesting a superior efficacy of maintained SMT compared to no treatment (88). Similarly, to assess the dose response of SMT for chronic LBP, patients were randomized to receive a variable number of SMT sessions out of a total of 18 visits over 6 weeks (91). When SMT was not applied, a light massage was used instead as an inactive control. In the short term, 12 SMT sessions were found to be the most efficacious, while in the long term, 18 visits with SMT yielded the greatest differences from the control (91). However, the results are limited by the fact that the control massage cannot be considered a true sham but rather a potentially active comparator. In contrast to these studies, a recent trial using sham cold laser treatment as a placebo did not find any differences with SMT or mobilization (93). This may seem like an appropriate sham, apparently devoid of any therapeutic effect. Interestingly, treatment expectancy was rated by all groups, and although there were no significant baseline differences, the sham cold laser group had the strongest relationship between expectations and pain relief (93). Research on placebo effects indicates that different types of placebos may hinder different outcomes, even via independent neurophysiological mechanisms (122). It is plausible that SMT and laser therapy may induce different placebo effects associated with distinct therapeutic rituals and expectations. Therefore, such a comparison may not be the best suited to answer this question concerning the efficacy of SMT for LBP.

Earlier Cochrane reviews on the effects of SMT for acute LBP concluded that SMT is not superior to inert or sham interventions (123), except when used in combination with other modalities, including exercise and patient education (26). A review for the American College of Physicians' guidelines found no effect over inert treatment for the management of acute LBP and only a small effect for chronic cases (111). Two different systematic reviews with meta-analyses specifically examined the differences in outcomes between SMT and sham manipulation (107, 108). Scholten-Peeters et al. reported a standardized mean difference (SMD) of −0.73 in favor of SMT for NP intensity on a visual analog or numerical rating scale immediately after treatment, and an SMD of −0.47 for LBP in the short term (107). Ruddock et al. found similar results (SMD of −0.36) in support of the efficacy of SMT for LBP intensity in the short term (108). Along the same lines, a recent network meta-analysis found that manual therapy (including SMT) significantly reduced pain and disability in the short and intermediate terms compared with inert treatment for acute and subacute LBP (109). Specifically, manual therapy was reported to be the most effective non-pharmacologic approach. However, the effects of manual therapy (including SMT) against sham treatment are still considered to be small and, more importantly, not clinically meaningful (110). The low quality of placebo interventions used for SMT trials may be partly to blame for the low quality of this evidence, the large degree of uncertainty, and the difficulty in drawing consistent conclusions (119).

Imperfect placebos are not uncommon in spine pain research and impact the quality of studies on other types of interventions (117). However, trials on SMT for spine pain most likely suffer from lower quality due to inherent difficulties in designing and applying a credible yet inert sham SMT treatment (124). It is therefore essential to improve the quality of SMT placebos for future studies to reduce the uncertainty regarding its efficacy for the management of spine pain.

## Discussion and Future Perspectives

Research on SM and SMT for the management of spine pain has progressed significantly in the past few years. Accumulating data provide evidence favoring the use of SMT in the management of acute, subacute, and chronic NP and LBP. The available clinical research suggests that SMT could be as effective as other conservative approaches used to treat non-specific and chronic primary spine pain. Nevertheless, this does not lead to consistent recommendations in the management of these conditions, and SMT often comes after advice/education and in combination with exercise. This probably suggests that the quality of evidence on the efficacy and effectiveness of SMT remains insufficient.

Accordingly, for the management of NP, recent guidelines recommend the use of SMT based mostly on consensus (75). In cases of recent onset (acute and subacute) NP, SMT is recommended before oral analgesics (75), although not muscle relaxants (74). Overall, clinical guidelines currently recommend SMT for the management of NP and cervical radiculopathy in combination with other approaches, particularly exercise and patient education (18, 74, 75).

For the management of LBP, most guidelines recommend SMT, with some discrepancies regarding the circumstances in which it should be administered (19, 125). For example, the United Kingdom's National Institute for Health and Care Excellence (NICE) guidelines make it imperative that SMT be offered alongside exercise therapy for LBP irrespective of the stage (113). In contrast, the American College of Physicians' guidelines endorse SMT as a frontline non-invasive intervention, partly because patients with acute LBP improve over time regardless of treatment (17). Specifically, for acute stages with or without radiculopathy, clinical practice guidelines recommend the addition of SMT to education, advice to remain active, and self-management (112, 114, 116). For chronic LBP, the guidelines tend to recommend the use of SMT either alone or preferably in combination with other approaches (frequently second to advice, education, and reassurance) for patients with or without leg pain (114, 115). Recently, a decision aid developed for managing chronic back pain by Canadian colleges of family physicians endorsed exercise and SMT as the only interventions for which benefits likely exceed harms (15). For low- and middle-income countries, the Global Spine Care Initiative produced guidelines taking into consideration practical aspects such as cost (18). Their recommendations are to consider the use of manual therapy (SMT and mobilizations) as one of the primary treatment options in patients with both acute and chronic spine pain and SMT specifically for radicular pain (18).

The recommendations for the use of SMT in patients with LBP and NP are mostly based on comparisons with other interventions, specifically, “recommended” interventions. Nevertheless, high-quality evidence indicates that SMT is not clinically superior to non-recommended interventions for the relief of chronic LBP (60). In fact, the main gap identified in clinical research on SMT for spine pain lies in the low quantity and quality of studies addressing its efficacy against inactive controls. Hence, the effects of SMT against placebo or sham SM remain uncertain. This parallels the state of research on most interventions for spine pain, as no treatment has been demonstrated to be superior to any other or to placebo (126–128). It could be argued that effective treatments for LBP and NP have a large share of non-specific effects. In order to understand what the specific effects of SMT are in future clinical trials on spine pain, the studies should include a placebo intervention that is indistinguishable from SM and that does not produce therapeutic effects (124). This can be achieved by determining the mechanisms of pain relief by SM and confirming that the placebo intervention does not influence these mechanisms. In addition, placebo interventions need to be validated by confirming that blinding was successful (129, 130).

Another important challenge for the immediate future of SMT research is the need to identify patients who will respond better to a trial of SMT. Research on clinical predictors of the response to SMT yielded mixed results (44, 131). It has been proposed that joint pain affecting multiple body regions may act as a moderator of the response to SMT. For example, in individuals with LBP, presenting NP complaints was associated with a decrease likelihood of responding to SMT (132). Similarly, the probability of benefitting from SMT for NP is reduced for patients presenting LBP complaints (133). Comorbidity is common in patients with chronic NP and LBP (134), with up to 50% of patients presenting symptoms in both regions (135). Patients with overlapping pain may represent a subgroup [i.e., non-localized LBP (136)]. It is also possible that chronic LBP and NP are different manifestations of the same disorder (137). This is compatible with the proposed definition of chronic primary pain. If this is the case, the effectiveness of SMT for spine pain in different regions should be similar, and the differences reported in the present study may reflect limitations of the current literature. Recently, more effort has been directed toward identifying biomechanical factors that may influence the response, including spinal stiffness and multifidus muscle involvement (138–141). The results have not always been consistent, although recent models that include demographic, clinical, biomechanical, and neurophysiological predictors are a promising avenue of research (132, 142). A better understanding of the specific effects of SMT via mechanistic research on specific subgroups of patients with high-quality designs that include validated placebo interventions is essential for future clinical research. This should translate into more homogenous recommendations on the use of SMT for specific patients, conditions, and pain states.

## Author Contributions

CG-M contributed to literature review and study selection and wrote the preliminary version of the manuscript. BP contributed to the literature review. MD contributed to manuscript editing. AO contributed to manuscript editing and guidance in its design. MP contributed to manuscript design, wrote the final version of the manuscript, and obtained funding. All authors have contributed significantly to this work and have read and approved the final version of the manuscript.

## Funding

All authors were supported by the Chaire de Recherche Internationale en Santé Neuromusculosquelettique. The contribution of Carlos Gevers-Montoro was supported by the Fonds de Recherche du Québec en Nature et Technologies (FRQNT) and the Asociación Española de Quiropráctica (AEQ). The contribution of Benjamin Provencher was supported by the Canadian Institutes of Health Research and the Fonds de Recherche du Québec en Santé (FRQS). The contribution of Mathieu Piché was also supported by the Fondation de Recherche en Chiropratique du Québec and the Fonds de Recherche du Québec en Santé (FRQS).

## Conflict of Interest

The authors declare that the research was conducted in the absence of any commercial or financial relationships that could be construed as a potential conflict of interest.

## Publisher's Note

All claims expressed in this article are solely those of the authors and do not necessarily represent those of their affiliated organizations, or those of the publisher, the editors and the reviewers. Any product that may be evaluated in this article, or claim that may be made by its manufacturer, is not guaranteed or endorsed by the publisher.

## Abbreviations

LBP: Low back pain
NP: Neck pain
SM: Spinal manipulation
SMT: Spinal manipulative therapy.

## References

[1] Manchikanti L Singh V Datta S Cohen SP Hirsch JA American Society of Interventional Pain P. Comprehensive review of epidemiology, scope, and impact of spinal pain. Pain Phys. (2009) 12:E35–70. 10.36076/ppj.2009/12/E35 19668291

[2] Hurwitz EL Randhawa K Yu H Cote P Haldeman S. The global spine care initiative: a summary of the global burden of low back and neck pain studies. Eur Spine J. (2018) 27:796–801. 10.1007/s00586-017-5432-9 29480409

[3] GBD Collaborators Disease I Incidence Prevalence Collaborators. Global, regional, national incidence. prevalence, and years lived with disability for 354 diseases and injuries for 195 countries and territories, 1990–2017: a systematic analysis for the global burden of disease study 2017. Lancet. (2018) 392:1789–858. 10.1016/S0140-6736(18)32279-7 30496104 PMC6227754

[4] Safiri S Kolahi AA Hoy D Buchbinder R Mansournia MA Bettampadi D. Global, regional, and national burden of neck pain in the general population, 1990-2017: systematic analysis of the global burden of disease study 2017. BMJ. (2020) 368:m791. 10.1136/bmj.m791 32217608 PMC7249252

[5] Wu A March L Zheng X Huang J Wang X Zhao J. Global low back pain prevalence and years lived with disability from 1990 to 2017: estimates from the global burden of disease study 2017. Ann Transl Med. (2020) 8:299. 10.21037/atm.2020.02.175 32355743 PMC7186678

[6] Hoy D Bain C Williams G March L Brooks P Blyth F. A systematic review of the global prevalence of low back pain. Arthritis Rheum. (2012) 64:2028–37. 10.1002/art.34347 22231424

[7] Breivik H Collett B Ventafridda V Cohen R Gallacher D. Survey of chronic pain in Europe: prevalence, impact on daily life, and treatment. Eur J Pain. (2006) 10:287–333. 10.1016/j.ejpain.2005.06.009 16095934

[8] Cohen SP Vase L Hooten WM. Chronic pain: an update on burden, best practices, new advances. Lancet. (2021) 397:2082–97. 10.1016/S0140-6736(21)00393-7 34062143

[9] Buchbinder R Underwood M Hartvigsen J Maher CG. The lancet series call to action to reduce low value care for low back pain: an update. Pain. (2020) 161 (Suppl. 1):S57–64. 10.1097/j.pain.0000000000001869 33090740 PMC7434211

[10] Bohnert AS Valenstein M Bair MJ Ganoczy D Mccarthy JF Ilgen MA. Association between opioid prescribing patterns and opioid overdose-related deaths. JAMA. (2011) 305:1315–21. 10.1001/jama.2011.370 21467284

[11] Gomes T Mamdani MM Dhalla IA Paterson JM Juurlink DN. Opioid dose and drug-related mortality in patients with nonmalignant pain. Arch Intern Med. (2011) 171:686–91. 10.1001/archinternmed.2011.117 21482846

[12] Ray WA Chung CP Murray KT Hall K Stein CM. Prescription of long-acting opioids and mortality in patients with chronic noncancer pain. JAMA. (2016) 315:2415–23. 10.1001/jama.2016.7789 27299617 PMC5030814

[13] Foster NE Anema JR Cherkin D Chou R Cohen SP Gross DP. Prevention and treatment of low back pain: evidence, challenges, promising directions. Lancet. (2018) 391:2368–83. 10.1016/S0140-6736(18)30489-6 29573872

[14] Corp N Mansell G Stynes S Wynne-Jones G Morso L Hill JC. Evidence-based treatment recommendations for neck and low back pain across Europe: a systematic review of guidelines. Eur J Pain. (2021) 25:275–95. 10.1002/ejp.1679 33064878 PMC7839780

[15] Kirkwood J Allan GM Korownyk CS Mccormack J Garrison S Thomas B. PEER simplified decision aid: chronic back pain treatment options in primary care. Can Fam Phys. (2021) 67:31–4. 10.46747/cfp.670131 33483394 PMC7822602

[16] Hurwitz EL Carragee EJ Van Der Velde G Carroll LJ Nordin M Guzman J. Treatment of neck pain: noninvasive interventions: results of the bone and joint decade 2000-2010 task force on neck pain and its associated disorders. Spine. (2008) 33:S123–52. 10.1097/BRS.0b013e3181644b1d 18204386

[17] Qaseem A Wilt TJ Mclean RM Forciea MA. Noninvasive treatments for acute, subacute, and chronic low back pain: a clinical practice guideline from the american college of physicians. Ann Intern Med. (2017) 166:514–30. 10.7326/M16-2367 28192789

[18] Chou R Cote P Randhawa K Torres P Yu H Nordin M. The global spine care initiative: applying evidence-based guidelines on the non-invasive management of back and neck pain to low- and middle-income communities. Eur Spine J. (2018) 27:851–60. 10.1007/s00586-017-5433-8 29460009

[19] Bailly F Trouvin AP Bercier S Dadoun S Deneuville JP Faguer R. Clinical guidelines and care pathway for management of low back pain with or without radicular pain. Joint Bone Spine. (2021) 88:105227. 10.1016/j.jbspin.2021.105227 34051387

[20] WHO. Guidelines on Basic Training and Safety in Chiropractic. >Geneva: World Health Organization (2005).

[21] Nelson CF Lawrence DJ Triano JJ Bronfort G Perle SM Metz RD. Chiropractic as spine care: a model for the profession. Chiropr Osteopat. (2005) 13:9. 10.1186/1746-1340-13-9 16000175 PMC1185558

[22] Schneider M Murphy D Hartvigsen J. Spine care as a framework for the chiropractic identity. J Chiropr Humanit. (2016) 23:14–21. 10.1016/j.echu.2016.09.004 27920614 PMC5127908

[23] Adams J Lauche R Peng W Steel A Moore C Amorin-Woods LG. A workforce survey of Australian chiropractic: the profile and practice features of a nationally representative sample of 2,005 chiropractors. BMC Complement Altern Med. (2017) 17:14. 10.1186/s12906-016-1542-x 28056964 PMC5217252

[24] Beliveau PJH Wong JJ Sutton DA Simon NB Bussières AE Mior SA. The chiropractic profession: a scoping review of utilization rates, reasons for seeking care, patient profiles, care provided. Chiroprac Man Ther. (2017) 25:35. 10.1186/s12998-017-0165-8 29201346 PMC5698931

[25] Herman PM Kommareddi M Sorbero ME Rutter CM Hays RD Hilton LG. Characteristics of chiropractic patients being treated for chronic low back and neck pain. J Manip Physiol Ther. (2018) 41:445–55. 10.1016/j.jmpt.2018.02.001 30121129 PMC6386466

[26] Walker BF French SD Grant W Green S. A cochrane review of combined chiropractic interventions for low-back pain. Spine. (2011) 36:230–42. 10.1097/BRS.0b013e318202ac73 21248591

[27] Deyo RA. The role of spinal manipulation in the treatment of low back pain. JAMA. (2017) 317:1418–9. 10.1001/jama.2017.3085 28399236

[28] Hurwitz EL. Epidemiology: spinal manipulation utilization. J Electromyogr Kinesiol. (2012) 22:648–54. 10.1016/j.jelekin.2012.01.006 22289432

[29] Whedon JM Haldeman S Petersen CL Schoellkopf W Mackenzie TA Lurie JD. Temporal trends and geographic variations in the supply of clinicians who provide spinal manipulation to medicare beneficiaries: a serial cross-sectional study. J Manip Physiol Ther. (2021) 44:177–85. 10.1016/j.jmpt.2021.02.002 33849727 PMC10695632

[30] Haavik H Kumari N Holt K Niazi IK Amjad I Pujari AN. The contemporary model of vertebral column joint dysfunction and impact of high-velocity, low-amplitude controlled vertebral thrusts on neuromuscular function. Eur J Appl Physiol. (2021) 121:2675–720. 10.1007/s00421-021-04727-z 34164712 PMC8416873

[31] Henderson CN. The basis for spinal manipulation: chiropractic perspective of indications and theory. J Electromyogr Kinesiol. (2012) 22:632–42. 10.1016/j.jelekin.2012.03.008 22513367

[32] Khodakarami N. Treatment of patients with low back pain: a comparison of physical therapy and chiropractic manipulation. Healthcare. (2020) 8:44. 10.3390/healthcare8010044 32102417 PMC7151187

[33] Coulter ID Herman PM Kommareddi M Hurwitz EL Shekelle PG. Measuring the appropriateness of spinal manipulation for chronic low back and chronic neck pain in chiropractic patients. Spine. (2021) 46:1344–53. 10.1097/BRS.0000000000004009 34517404 PMC8438222

[34] Meeker WC Haldeman S. Chiropractic: a profession at the crossroads of mainstream and alternative medicine. Ann Intern Med. (2002) 136:216–27. 10.7326/0003-4819-136-3-200202050-00010 11827498

[35] Fritz JM Cleland J. Effectiveness versus efficacy: more than a debate over language. J Orthop Sports Phys Ther. (2003) 33:163–5. 10.2519/jospt.2003.33.4.163 12723672

[36] Borghouts JA Koes BW Bouter LM. The clinical course and prognostic factors of non-specific neck pain: a systematic review. Pain. (1998) 77:1–13. 10.1016/S0304-3959(98)00058-X 9755013

[37] Coulter ID Crawford C Vernon H Hurwitz EL Khorsan R Booth MS. Manipulation and mobilization for treating chronic nonspecific neck pain: a systematic review and meta-analysis for an appropriateness panel. Pain Phys. (2019) 22:E55–70. 10.36076/ppj/2019.22.E55 30921975 PMC6800035

[38] Binder AI. Neck pain. BMJ Clin Evid. (2008) 2008:1103. PMC2907992 19445809

[39] Nicholas M Vlaeyen JWS Rief W Barke A Aziz Q Benoliel R. The IASP classification of chronic pain for ICD-11: chronic primary pain. Pain. (2019) 160:28–37. 10.1097/j.pain.0000000000001390 30586068

[40] Treede RD Rief W Barke A Aziz Q Bennett MI Benoliel R. Chronic pain as a symptom or a disease: the IASP classification of chronic pain for the international classification of diseases (ICD-11). Pain. (2019) 160:19–27. 10.1097/j.pain.0000000000001384 30586067

[41] Suvarnnato T Puntumetakul R Kaber D Boucaut R Boonphakob Y Arayawichanon P. The effects of thoracic manipulation versus mobilization for chronic neck pain: a randomized controlled trial pilot study. J Phys Ther Sci. (2013) 25:865–71. 10.1589/jpts.25.865 24259872 PMC3820396

[42] Masaracchio M Kirker K States R Hanney WJ Liu X Kolber M. Thoracic spine manipulation for the management of mechanical neck pain: a systematic review and meta-analysis. PLoS ONE. (2019) 14:e0211877. 10.1371/journal.pone.0211877 30759118 PMC6373960

[43] Gonzalez-Iglesias J Fernandez-De-Las-Penas C Cleland JA Gutierrez-Vega Mdel R. Thoracic spine manipulation for the management of patients with neck pain: a randomized clinical trial. J Orthop Sports Phys Ther. (2009) 39:20–7. 10.2519/jospt.2009.2914 19209478

[44] Cleland JA Mintken PE Carpenter K Fritz JM Glynn P Whitman J. Examination of a clinical prediction rule to identify patients with neck pain likely to benefit from thoracic spine thrust manipulation and a general cervical range of motion exercise: multi-center randomized clinical trial. Phys Ther. (2010) 90:1239–50. 10.2522/ptj.20100123 20634268

[45] Lau HM Wing Chiu TT Lam TH. The effectiveness of thoracic manipulation on patients with chronic mechanical neck pain - a randomized controlled trial. Man Ther. (2011) 16:141–7. 10.1016/j.math.2010.08.003 20813577

[46] Bronfort G Evans R Anderson AV Svendsen KH Bracha Y Grimm RH. Spinal manipulation, medication, or home exercise with advice for acute and subacute neck pain: a randomized trial. Ann Intern Med. (2012) 156:1–10. 10.7326/0003-4819-156-1-201201030-00002 22213489

[47] Evans R Bronfort G Schulz C Maiers M Bracha Y Svendsen K. Supervised exercise with and without spinal manipulation performs similarly and better than home exercise for chronic neck pain: a randomized controlled trial. Spine. (2012) 37:903–14. 10.1097/BRS.0b013e31823b3bdf 22024905

[48] Saavedra-Hernandez M Castro-Sanchez AM Arroyo-Morales M Cleland JA Lara-Palomo IC Fernandez-De-Las-Penas C. Short-term effects of kinesio taping versus cervical thrust manipulation in patients with mechanical neck pain: a randomized clinical trial. J Orthop Sports Phys Ther. (2012) 42:724–30. 10.2519/jospt.2012.4086 22523090

[49] Gorrell LM Beath K Engel RM. Manual and instrument applied cervical manipulation for mechanical neck pain: a randomized controlled trial. J Manip Physiol Ther. (2016) 39:319–29. 10.1016/j.jmpt.2016.03.003 27180949

[50] Galindez-Ibarbengoetxea X Setuain I Ramirez-Velez R Andersen LL Gonzalez-Izal M Jauregi A. Short-term effects of manipulative treatment versus a therapeutic home exercise protocol for chronic cervical pain: a randomized clinical trial. J Back Musculoskelet Rehabil. (2018) 31:133–45. 10.3233/BMR-169723 28826170

[51] Gemmell H Miller P. Relative effectiveness and adverse effects of cervical manipulation, mobilisation and the activator instrument in patients with sub-acute non-specific neck pain: results from a stopped randomised trial. Chiropr Osteopat. (2010) 18:20. 10.1186/1746-1340-18-20 20618936 PMC2927873

[52] Dunning JR Cleland JA Waldrop MA Arnot CF Young IA Turner M. Upper cervical and upper thoracic thrust manipulation versus nonthrust mobilization in patients with mechanical neck pain: a multicenter randomized clinical trial. J Orthop Sports Phys Ther. (2012) 42:5–18. 10.2519/jospt.2012.3894 21979312

[53] Masaracchio M Cleland JA Hellman M Hagins M. Short-term combined effects of thoracic spine thrust manipulation and cervical spine nonthrust manipulation in individuals with mechanical neck pain: a randomized clinical trial. J Orthop Sports Phys Ther. (2013) 43:118–27. 10.2519/jospt.2013.4221 23221367

[54] Izquierdo Perez H Alonso Perez JL Gil Martinez A La Touche R Lerma-Lara S Commeaux Gonzalez N. Is one better than another?: a randomized clinical trial of manual therapy for patients with chronic neck pain. Man Ther. (2014) 19:215–21. 10.1016/j.math.2013.12.002 24467843

[55] Salom-Moreno J Ortega-Santiago R Cleland JA Palacios-Cena M Truyols-Dominguez S Fernandez-De-Las-Penas C. Immediate changes in neck pain intensity and widespread pressure pain sensitivity in patients with bilateral chronic mechanical neck pain: a randomized controlled trial of thoracic thrust manipulation vs non-thrust mobilization. J Manip Physiol Ther. (2014) 37:312–9. 10.1016/j.jmpt.2014.03.003 24880778

[56] Alonso-Perez JL Lopez-Lopez A La Touche R Lerma-Lara S Suarez E Rojas J. Hypoalgesic effects of three different manual therapy techniques on cervical spine and psychological interaction: a randomized clinical trial. J Bodyw Mov Ther. (2017) 21:798–803. 10.1016/j.jbmt.2016.12.005 29037630

[57] Griswold D Learman K Kolber MJ O'halloran B Cleland JA. Pragmatically applied cervical and thoracic nonthrust manipulation versus thrust manipulation for patients with mechanical neck pain: a multicenter randomized clinical trial. J Orthop Sports Phys Ther. (2018) 48:137–45. 10.2519/jospt.2018.7738 29406835

[58] Joshi S Balthillaya G Neelapala YVR. Immediate effects of cervicothoracic junction mobilization versus thoracic manipulation on the range of motion and pain in mechanical neck pain with cervicothoracic junction dysfunction: a pilot randomized controlled trial. Chiropr Man Therap. (2020) 28:38. 10.1186/s12998-020-00327-4 32762708 PMC7412667

[59] Gross A Langevin P Burnie SJ Bedard-Brochu MS Empey B Dugas E. Manipulation and mobilisation for neck pain contrasted against an inactive control or another active treatment. Cochrane Database Syst Rev. (2015) 9:CD004249. 10.1002/14651858.CD004249.pub4 26397370 PMC10883412

[60] Rubinstein SM De Zoete A Van Middelkoop M Assendelft WJJ De Boer MR Van Tulder MW. Benefits and harms of spinal manipulative therapy for the treatment of chronic low back pain: systematic review and meta-analysis of randomised controlled trials. BMJ. (2019) 364:l689. 10.1136/bmj.l689 30867144 PMC6396088

[61] Bolton PS Budgell BS. Spinal manipulation and spinal mobilization influence different axial sensory beds. Med Hypotheses. (2006) 66:258–62. 10.1016/j.mehy.2005.08.054 16242852

[62] Cross KM Kuenze C Grindstaff TL Hertel J. Thoracic spine thrust manipulation improves pain, range of motion, and self-reported function in patients with mechanical neck pain: a systematic review. J Orthop Sports Phys Ther. (2011) 41:633–42. 10.2519/jospt.2011.3670 21885904

[63] Saavedra-Hernandez M Arroyo-Morales M Cantarero-Villanueva I Fernandez-Lao C Castro-Sanchez AM Puentedura EJ. Short-term effects of spinal thrust joint manipulation in patients with chronic neck pain: a randomized clinical trial. Clin Rehabil. (2013) 27:504–12. 10.1177/0269215512464501 23129812

[64] Vincent K Maigne JY Fischhoff C Lanlo O Dagenais S. Systematic review of manual therapies for nonspecific neck pain. Joint Bone Spine. (2013) 80:508–15. 10.1016/j.jbspin.2012.10.006 23165183

[65] Young JL Walker D Snyder S Daly K. Thoracic manipulation versus mobilization in patients with mechanical neck pain: a systematic review. J Man Manip Ther. (2014) 22:141–53. 10.1179/2042618613Y.0000000043 25125936 PMC4101553

[66] Haas M Bronfort G Evans R Schulz C Vavrek D Takaki L. Dose-response and efficacy of spinal manipulation for care of cervicogenic headache: a dual-center randomized controlled trial. Spine J. (2018) 18:1741–54. 10.1016/j.spinee.2018.02.019 29481979 PMC6107442

[67] Pasquier M Daneau C Marchand AA Lardon A Descarreaux M. Spinal manipulation frequency and dosage effects on clinical and physiological outcomes: a scoping review. Chiropr Man Therap. (2019) 27:23. 10.1186/s12998-019-0244-0 31139346 PMC6530068

[68] Southerst D Nordin MC Cote P Shearer HM Varatharajan S Yu H. Is exercise effective for the management of neck pain and associated disorders or whiplash-associated disorders? A systematic review by the Ontario protocol for traffic injury management (OPTIMa) collaboration. Spine J. (2016) 16:1503–23. 10.1016/j.spinee.2014.02.014 24534390

[69] D'sylva J Miller J Gross A Burnie SJ Goldsmith CH Graham N. Manual therapy with or without physical medicine modalities for neck pain: a systematic review. Man Ther. (2010) 15:415–33. 10.1016/j.math.2010.04.003 20538501

[70] Miller J Gross A D'sylva J Burnie SJ Goldsmith CH Graham N. Manual therapy and exercise for neck pain: a systematic review. Man Ther. (2010) 15:334–54. 10.1016/j.math.2010.02.007 20593537

[71] Fredin K Loras H. Manual therapy, exercise therapy or combined treatment in the management of adult neck pain - a systematic review and meta-analysis. Musculoskelet Sci Pract. (2017) 31:62–71. 10.1016/j.msksp.2017.07.005 28750310

[72] Hidalgo B Hall T Bossert J Dugeny A Cagnie B Pitance L. The efficacy of manual therapy and exercise for treating non-specific neck pain: a systematic review. J Back Musculoskelet Rehabil. (2017) 30:1149–69. 10.3233/BMR-169615 28826164 PMC5814665

[73] Wong JJ Shearer HM Mior S Jacobs C Cote P Randhawa K. Are manual therapies, passive physical modalities, or acupuncture effective for the management of patients with whiplash-associated disorders or neck pain and associated disorders? An update of the bone and joint decade task force on neck pain and its associated disorders by the OPTIMa collaboration. Spine J. (2016) 16:1598–630. 10.1016/j.spinee.2015.08.024 26707074

[74] Cote P Wong JJ Sutton D Shearer HM Mior S Randhawa K. Management of neck pain and associated disorders: a clinical practice guideline from the Ontario protocol for traffic injury management (OPTIMa) collaboration. Eur Spine J. (2016) 25:2000–22. 10.1007/s00586-016-4467-7 26984876

[75] Kjaer P Kongsted A Hartvigsen J Isenberg-Jorgensen A Schiottz-Christensen B Soborg B. National clinical guidelines for non-surgical treatment of patients with recent onset neck pain or cervical radiculopathy. Eur Spine J. (2017) 26:2242–57. 10.1007/s00586-017-5121-8 28523381

[76] Vlaeyen JWS Maher CG Wiech K Van Zundert J Meloto CB Diatchenko L. Low back pain. Nat Rev Dis Primers. (2018) 4:52. 10.1038/s41572-018-0052-1 30546064

[77] Finley CR Chan DS Garrison S Korownyk C Kolber MR Campbell S. What are the most common conditions in primary care? Systematic review. Can Fam Phys. (2018) 64:832–40. Available online at: https://www.cfp.ca/content/64/11/832 30429181 PMC6234945

[78] Hartvigsen J Hancock MJ Kongsted A Louw Q Ferreira ML Genevay S. What low back pain is and why we need to pay attention. Lancet. (2018) 391:2356–67. 10.1016/S0140-6736(18)30480-X 29573870

[79] Juni P Battaglia M Nuesch E Hammerle G Eser P Van Beers R. A randomised controlled trial of spinal manipulative therapy in acute low back pain. Ann Rheum Dis. (2009) 68:1420–7. 10.1136/ard.2008.093757 18775942

[80] Cecchi F Molino-Lova R Chiti M Pasquini G Paperini A Conti AA. Spinal manipulation compared with back school and with individually delivered physiotherapy for the treatment of chronic low back pain: a randomized trial with one-year follow-up. Clin Rehabil. (2010) 24:26–36. 10.1177/0269215509342328 20053720

[81] Bronfort G Maiers MJ Evans RL Schulz CA Bracha Y Svendsen KH. Supervised exercise, spinal manipulation, and home exercise for chronic low back pain: a randomized clinical trial. Spine J. (2011) 11:585–98. 10.1016/j.spinee.2011.01.036 21622028

[82] Petersen T Larsen K Nordsteen J Olsen S Fournier G Jacobsen S. The McKenzie method compared with manipulation when used adjunctive to information and advice in low back pain patients presenting with centralization or peripheralization: a randomized controlled trial. Spine. (2011) 36:1999–2010. 10.1097/BRS.0b013e318201ee8e 21358492

[83] Goertz CM Long CR Hondras MA Petri R Delgado R Lawrence DJ. Adding chiropractic manipulative therapy to standard medical care for patients with acute low back pain: results of a pragmatic randomized comparative effectiveness study. Spine. (2013) 38:627–34. 10.1097/BRS.0b013e31827733e7 23060056

[84] Bronfort G Hondras MA Schulz CA Evans RL Long CR Grimm R. Spinal manipulation and home exercise with advice for subacute and chronic back-related leg pain: a trial with adaptive allocation. Ann Intern Med. (2014) 161:381–91. 10.7326/M14-0006 25222385

[85] Schneider M Haas M Glick R Stevans J Landsittel D. Comparison of spinal manipulation methods and usual medical care for acute and subacute low back pain: a randomized clinical trial. Spine. (2015) 40:209–17. 10.1097/BRS.0000000000000724 25423308 PMC4326596

[86] Nambi G Kamal W Es S Joshi S Trivedi P. Spinal manipulation plus laser therapy versus laser therapy alone in the treatment of chronic non-specific low back pain: a randomized controlled study. Eur J Phys Rehabil Med. (2018) 54:880–9. 10.23736/S1973-9087.18.05005-0 29687966

[87] Ghasabmahaleh SH Rezasoltani Z Dadarkhah A Hamidipanah S Mofrad RK Najafi S. Spinal manipulation for subacute and chronic lumbar radiculopathy: a randomized controlled Trial. Am J Med. (2021) 134:135–41. 10.1016/j.amjmed.2020.08.005 32931763

[88] Senna MK Machaly SA. Does maintained spinal manipulation therapy for chronic nonspecific low back pain result in better long-term outcome? Spine. (2011) 36:1427–37. 10.1097/BRS.0b013e3181f5dfe0 21245790

[89] Von Heymann WJ Schloemer P Timm J Muehlbauer B. Spinal high-velocity low amplitude manipulation in acute nonspecific low back pain: a double-blinded randomized controlled trial in comparison with diclofenac and placebo. Spine. (2013) 38:540–8. 10.1097/BRS.0b013e318275d09c 23026869

[90] Bialosky JE George SZ Horn ME Price DD Staud R Robinson ME. Spinal manipulative therapy-specific changes in pain sensitivity in individuals with low back pain (NCT01168999). J Pain. (2014) 15:136–48. 10.1016/j.jpain.2013.10.005 24361109 PMC3946602

[91] Haas M Vavrek D Peterson D Polissar N Neradilek MB. Dose-response and efficacy of spinal manipulation for care of chronic low back pain: a randomized controlled trial. Spine J. (2014) 14:1106–16. 10.1016/j.spinee.2013.07.468 24139233 PMC3989479

[92] Vieira-Pellenz F Oliva-Pascual-Vaca A Rodriguez-Blanco C Heredia-Rizo AM Ricard F Almazan-Campos G. Short-term effect of spinal manipulation on pain perception, spinal mobility, and full height recovery in male subjects with degenerative disk disease: a randomized controlled trial. Arch Phys Med Rehabil. (2014) 95:1613–9. 10.1016/j.apmr.2014.05.002 24862763

[93] Thomas JS Clark BC Russ DW France CR Ploutz-Snyder R Corcos DM. Effect of spinal manipulative and mobilization therapies in young adults with mild to moderate chronic low back pain: a randomized clinical trial. JAMA Netw Open. (2020) 3:e2012589. 10.1001/jamanetworkopen.2020.12589 32756930 PMC7407093

[94] Hondras MA Long CR Cao Y Rowell RM Meeker WC. A randomized controlled trial comparing 2 types of spinal manipulation and minimal conservative medical care for adults 55 years and older with subacute or chronic low back pain. J Manipulative Physiol Ther. (2009) 32:330–43. 10.1016/j.jmpt.2009.04.012 19539115

[95] Cook C Learman K Showalter C Kabbaz V O'halloran B. Early use of thrust manipulation versus non-thrust manipulation: a randomized clinical trial. Man Ther. (2013) 18:191–8. 10.1016/j.math.2012.08.005 23040656

[96] Xia T Long CR Gudavalli MR Wilder DG Vining RD Rowell RM. Similar effects of thrust and nonthrust spinal manipulation found in adults with subacute and chronic low back pain: a controlled trial with adaptive allocation. Spine. (2016) 41:E702–9. 10.1097/BRS.0000000000001373 26656041 PMC4902754

[97] Coulter ID Crawford C Hurwitz EL Vernon H Khorsan R Suttorp Booth M. Manipulation and mobilization for treating chronic low back pain: a systematic review and meta-analysis. Spine J. (2018) 18:866–79. 10.1016/j.spinee.2018.01.013 29371112 PMC6020029

[98] Paige NM Miake-Lye IM Booth MS Beroes JM Mardian AS Dougherty P. Association of spinal manipulative therapy with clinical benefit and harm for acute low back pain: systematic review and meta-analysis. JAMA. (2017) 317:1451–60. 10.1001/jama.2017.3086 28399251 PMC5470352

[99] Hidalgo B Detrembleur C Hall T Mahaudens P Nielens H. The efficacy of manual therapy and exercise for different stages of non-specific low back pain: an update of systematic reviews. J Man Manip Ther. (2014) 22:59–74. 10.1179/2042618613Y.0000000041 24976749 PMC4017797

[100] Van Middelkoop M Rubinstein SM Verhagen AP Ostelo RW Koes BW Van Tulder M. W. Exercise therapy for chronic nonspecific low-back pain. Best Pract Res Clin Rheumatol. (2010) 24:193–204. 10.1016/j.berh.2010.01.002 20227641

[101] Owen PJ Miller CT Mundell NL Verswijveren S Tagliaferri SD Brisby H. Which specific modes of exercise training are most effective for treating low back pain? Network meta-analysis. Br J Sports Med. (2020) 54:1279–87. 10.1136/bjsports-2019-100886 31666220 PMC7588406

[102] Standaert CJ Friedly J Erwin MW Lee MJ Rechtine G Henrikson NB. Comparative effectiveness of exercise, acupuncture, and spinal manipulation for low back pain. Spine. (2011) 36:S120–30. 10.1097/BRS.0b013e31822ef878 21952184

[103] De Zoete A De Boer MR Rubinstein SM Van Tulder MW Underwood M Hayden JA. Moderators of the effect of spinal manipulative therapy on pain relief and function in patients with chronic low back pain: an individual participant data meta-analysis. Spine. (2021) 46:E505–17. 10.1097/BRS.0000000000003814 33186277 PMC7993913

[104] De Zoete A Rubinstein S De Boer M Ostelo R Underwood M Hayden J. The effect of spinal manipulative therapy on pain relief and function in patients with chronic low back pain: an individual participant data meta-analysis. Physiotherapy. (2021) 112:121–34. 10.1016/j.physio.2021.03.006 34049207

[105] Goertz CM Pohlman KA Vining RD Brantingham JW Long CR. Patient-centered outcomes of high-velocity, low-amplitude spinal manipulation for low back pain: a systematic review. J Electromyogr Kinesiol. (2012) 22:670–91. 10.1016/j.jelekin.2012.03.006 22534288

[106] Blanchette MA Stochkendahl MJ Borges Da Silva R Boruff J Harrison P Bussieres A. Effectiveness and economic evaluation of chiropractic care for the treatment of low back pain: a systematic review of pragmatic studies. PLoS ONE. (2016) 11:e0160037. 10.1371/journal.pone.0160037 27487116 PMC4972425

[107] Scholten-Peeters GG Thoomes E Konings S Beijer M Verkerk K Koes BW. Is manipulative therapy more effective than sham manipulation in adults : a systematic review and meta-analysis. Chiropr Man Therap. (2013) 21:34. 10.1186/2045-709X-21-34 24274314 PMC3850908

[108] Ruddock JK Sallis H Ness A Perry RE. Spinal manipulation vs sham manipulation for nonspecific low back pain: a systematic review and meta-analysis. J Chiropr Med. (2016) 15:165–83. 10.1016/j.jcm.2016.04.014 27660593 PMC5021904

[109] Gianola S Bargeri S Del Castillo G Corbetta D Turolla A Andreano A. Effectiveness of treatments for acute and subacute mechanical non-specific low back pain: a systematic review with network meta-analysis. Br J Sports Med. (2021) 1–11. 10.1136/bjsports-2020-103596 33849907 PMC8685632

[110] Lavazza C Galli M Abenavoli A Maggiani A. Sham treatment effects in manual therapy trials on back pain patients: a systematic review and pairwise meta-analysis. BMJ Open. (2021) 11:e045106. 10.1136/bmjopen-2020-045106 33947735 PMC8098952

[111] Chou R Deyo R Friedly J Skelly A Hashimoto R Weimer M. Nonpharmacologic therapies for low back pain: a systematic review for an american college of physicians clinical practice guideline. Ann Intern Med. (2017) 166:493–505. 10.7326/M16-2459 28192793

[112] Dagenais S Tricco AC Haldeman S. Synthesis of recommendations for the assessment and management of low back pain from recent clinical practice guidelines. Spine J. (2010) 10:514–29. 10.1016/j.spinee.2010.03.032 20494814

[113] Bernstein IA Malik Q Carville S Ward S. Low back pain and sciatica: summary of NICE guidance. BMJ. (2017) 356:i6748. 10.1136/bmj.i6748 28062522

[114] Wong JJ Cote P Sutton DA Randhawa K Yu H Varatharajan S. Clinical practice guidelines for the noninvasive management of low back pain: a systematic review by the Ontario protocol for traffic injury management (OPTIMa) collaboration. Eur J Pain. (2017) 21:201–16. 10.1002/ejp.931 27712027

[115] Bussieres AE Stewart G Al-Zoubi F Decina P Descarreaux M Haskett D. Spinal manipulative therapy and other conservative treatments for low back pain: a guideline from the canadian chiropractic guideline initiative. J Manip Physiol Ther. (2018) 41:265–93. 10.1016/j.jmpt.2017.12.004 29606335

[116] Stochkendahl MJ Kjaer P Hartvigsen J Kongsted A Aaboe J Andersen M. National clinical guidelines for non-surgical treatment of patients with recent onset low back pain or lumbar radiculopathy. Eur Spine J. (2018) 27:60–75. 10.1007/s00586-017-5099-2 28429142

[117] Machado LA Kamper SJ Herbert RD Maher CG Mcauley JH. Imperfect placebos are common in low back pain trials: a systematic review of the literature. Eur Spine J. (2008) 17:889–904. 10.1007/s00586-008-0664-3 18421484 PMC2443262

[118] Hancock MJ Maher CG Latimer J Mcauley JH. Selecting an appropriate placebo for a trial of spinal manipulative therapy. Aust J Physiother. (2006) 52:135–8. 10.1016/S0004-9514(06)70049-6 16764551

[119] Puhl AA Reinhart CJ Doan JB Vernon H. The quality of placebos used in randomized, controlled trials of lumbar and pelvic joint thrust manipulation-a systematic review. Spine J. (2017) 17:445–56. 10.1016/j.spinee.2016.11.003 27888138

[120] Cormier S Lavigne GL Choiniere M Rainville P. Expectations predict chronic pain treatment outcomes. Pain. (2016) 157:329–38. 10.1097/j.pain.0000000000000379 26447703

[121] Vernon H Puhl A Reinhart C. Systematic review of clinical trials of cervical manipulation: control group procedures and pain outcomes. Chiropr Man Therap. (2011) 19:3. 10.1186/2045-709X-19-3 21247413 PMC3039829

[122] Benedetti F Dogue S. Different placebos, different mechanisms, different outcomes: lessons for clinical trials. PLoS ONE. (2015) 10:e0140967. 10.1371/journal.pone.0140967 26536471 PMC4633056

[123] Rubinstein SM Terwee CB Assendelft WJ De Boer MR Van Tulder MW. Spinal manipulative therapy for acute low back pain: an update of the cochrane review. Spine. (2013) 38:E158–77. 10.1097/BRS.0b013e31827dd89d 23169072

[124] Gevers-Montoro C Provencher B Descarreaux M Ortega De Mues A Piche M. Neurophysiological mechanisms of chiropractic spinal manipulation for spine pain. Eur J Pain. (2021) 25:1429–48. 10.1002/ejp.1773 33786932

[125] Oliveira CB Maher CG Pinto RZ Traeger AC Lin CC Chenot JF. Clinical practice guidelines for the management of non-specific low back pain in primary care: an updated overview. Eur Spine J. (2018) 27:2791–803. 10.1007/s00586-018-5673-2 29971708

[126] Machado LA Kamper SJ Herbert RD Maher CG Mcauley JH. Analgesic effects of treatments for non-specific low back pain: a meta-analysis of placebo-controlled randomized trials. Rheumatology. (2009) 48:520–7. 10.1093/rheumatology/ken470 19109315

[127] Artus M Van Der Windt DA Jordan KP Hay EM. Low back pain symptoms show a similar pattern of improvement following a wide range of primary care treatments: a systematic review of randomized clinical trials. Rheumatology. (2010) 49:2346–56. 10.1093/rheumatology/keq245 20713495

[128] Van Lennep J Trossel F Perez R Otten RHJ Van Middendorp H Evers AWM. Placebo effects in low back pain: a systematic review and meta-analysis of the literature. Eur J Pain. (2021) 25:1876–97. 10.1002/ejp.1811 34051018 PMC8518410

[129] Vernon H Triano JJ Ross JK Tran SK Soave DM Dinulos MD. Validation of a novel sham cervical manipulation procedure. Spine J. (2012) 12:1021–8. 10.1016/j.spinee.2012.10.009 23158966 PMC3513586

[130] Chaibi A Saltyte Benth J Bjorn Russell M. Validation of placebo in a manual therapy randomized controlled trial. Sci Rep. (2015) 5:11774. 10.1038/srep11774 26145718 PMC4491841

[131] Hancock MJ Maher CG Latimer J Herbert RD Mcauley JH. Independent evaluation of a clinical prediction rule for spinal manipulative therapy: a randomised controlled trial. Eur Spine J. (2008) 17:936–43. 10.1007/s00586-008-0679-9 18427840 PMC2443269

[132] Hadizadeh M Kawchuk GN Prasad N Fritz JM. Predicting who responds to spinal manipulative therapy using a short-time frame methodology: results from a 238-participant study. PLoS ONE. (2020) 15:e0242831. 10.1371/journal.pone.0242831 33232379 PMC7685475

[133] Schellingerhout JM Verhagen AP Heymans MW Pool JJM Vonk F Koes BW. Which subgroups of patients with non-specific neck pain are more likely to benefit from spinal manipulation therapy, physiotherapy, or usual care? Pain. (2008) 139:670–80. 10.1016/j.pain.2008.07.015 18774225

[134] Guez M Hildingsson C Nasic S Toolanen G. Chronic low back pain in individuals with chronic neck pain of traumatic and non-traumatic origin: a population-based study. Acta Orthop. (2006) 77:132–7. 10.1080/17453670610045812 16534713

[135] Overas CK Johansson MS De Campos TF Ferreira ML Natvig B Mork PJ. Distribution and prevalence of musculoskeletal pain co-occurring with persistent low back pain: a systematic review. BMC Musculoskelet Disord. (2021) 22:91. 10.1186/s12891-020-03893-z 33461514 PMC7814622

[136] Coggon D Ntani G Walker-Bone K Palmer KT Felli VE Harari R. Epidemiological differences between localized and nonlocalized low back pain. Spine. (2017) 42:740–7. 10.1097/BRS.0000000000001956 27820794 PMC5418102

[137] Leboeuf-Yde C Fejer R Nielsen J Kyvik KO Hartvigsen J. Pain in the three spinal regions: the same disorder? Data from a population-based sample of 34,902 Danish adults. Chiropr Man Therap. (2012) 20:11. 10.1186/2045-709X-20-11 22480304 PMC3368748

[138] Fritz JM Koppenhaver SL Kawchuk GN Teyhen DS Hebert JJ Childs JD. Preliminary investigation of the mechanisms underlying the effects of manipulation: exploration of a multivariate model including spinal stiffness, multifidus recruitment, clinical findings. Spine. (2011) 36:1772–81. 10.1097/BRS.0b013e318216337d 21358568 PMC3150636

[139] Koppenhaver SL Fritz JM Hebert JJ Kawchuk GN Childs JD Parent EC. Association between changes in abdominal and lumbar multifidus muscle thickness and clinical improvement after spinal manipulation. J Orthop Sports Phys Ther. (2011) 41:389–99. 10.2519/jospt.2011.3632 21471653

[140] Wong AY Parent EC Dhillon SS Prasad N Kawchuk GN. Do participants with low back pain who respond to spinal manipulative therapy differ biomechanically from nonresponders, untreated controls or asymptomatic controls? Spine. (2015) 40:1329–37. 10.1097/BRS.0000000000000981 26020851

[141] Page I Descarreaux M. Effects of spinal manipulative therapy biomechanical parameters on clinical and biomechanical outcomes of participants with chronic thoracic pain: a randomized controlled experimental trial. BMC Musculoskelet Disord. (2019) 20:29. 10.1186/s12891-019-2408-4 30658622 PMC6339327

[142] Nim CG Kawchuk GN Schiottz-Christensen B O'neill S. Changes in pain sensitivity and spinal stiffness in relation to responder status following spinal manipulative therapy in chronic low back pain: a secondary explorative analysis of a randomized trial. BMC Musculoskelet Disord. (2021) 22:23. 10.1186/s12891-020-03873-3 33407345 PMC7786943

